# Increasing serum miR-409-3p predicts the major adverse cardiac adverse events in elderly patients after hip fracture surgery

**DOI:** 10.1186/s12891-023-07049-7

**Published:** 2023-11-28

**Authors:** Zhengtao Sun, Kai Cheng, Guochao Jin, Jian Jia

**Affiliations:** Department of Osteoarticular Surgery, Linfen People’s Hospital, No.319 Gulou West Street, Yao Du District, Linfen, 041000 China

**Keywords:** Hip fracture repair surgery, Postoperative complication, Cardiac and renal function, Major adverse cardiovascular disease

## Abstract

**Background:**

Major adverse cardiovascular events (MACE) are critical complications responsible for the morbidity and mortality of elderly hip fracture patients. There was an urgent need to explore an effect biomarker for predicting MACE in elderly patients receiving hip fracture surgery.

**Objective:**

This study focused on an age-related miRNA, miR-409-3p, and assessed its significance in elderly hip fracture patients.

**Methods:**

A total of 267 hip fracture patients were enrolled in this study including 104 elderly patients (age ≥ 60 years). All patients were followed up for 1 year to monitor the occurrence of MACE. The risk factors for the occurrence of MACE were evaluated by the logistic regression analysis.

**Results:**

Elderly age and reduced cardiac and renal function were identified as risk factors for MACE in hip fracture patients. Elderly patients also showed a high incidence of MACE. In elderly hip fracture patients, significant upregulation of miR-409-3p was observed, which was associated with patients’ elderly age, higher level of revised cardiac risk index (RCRI), lower left ventricular ejection fraction (LVEF), and higher levels of N-terminal pro-brain natriuretic peptide (NT-proBNP), creatine kinase-MB (CK-MB), and high sensitivity troponin I (hsTnI). Additionally, miR-409-3p was identified as an independent factor for the MACE in elderly patients received hip fracture surgery.

**Conclusion:**

Upregulated miR-409-3p was an age-related miRNA and could predict the occurrence of MACE in elderly hip fracture patients.

**Supplementary Information:**

The online version contains supplementary material available at 10.1186/s12891-023-07049-7.

## Introduction

The number of elderly people with hip fractures is gradually increasing year by year due to the accelerating aging of the population [1]. Surgical therapy could effectively alleviate pain and reduce the occurrence of complications and shorten the time in bed after the surgery [2]. Due to the complicated basic diseases, the prognosis of elderly patients who received hip fracture surgery is poor [3]. It was reported that the postoperative mortality rate of elderly patients receiving hip surgery was as high as 20-40%, where less than half of the survival patients could return to their pre-injury status [4]. The mortality is high within 1 year after surgery, and then the risk gradually decreases [5]. Previous studies have analyzed the independent factors for the postoperative mortality of hip fracture surgery, and aging, complications, and deferred operation were identified [6–8]. Major adverse cardiovascular events (MACE) are one of the most common complications and are important adverse prognostic factors for hip fracture surgery, which dramatically affects the postoperative life of elderly patients. Elderly patients are prone to MACE due to pathophysiological changes, such as reduced cardiac reserve capacity and degenerative changes in heart structure [9]. Hip fracture induced traumatic stress, pain, and other stimulations, which increase the risk of MACE, especially in the perioperative period [10]. The risk factors for MACE might be different from various research conditions and study subjects, and it is necessary to explore effect biomarkers that could predict MACE in elderly patients who received hip fracture surgery.

Thanks to the great progression made in molecular biology in the past decades, the significance of non-coding RNAs (ncRNAs) in human disease development has attracted huge attention [11]. Some studies identified aging-related ncRNAs that regulate cardiac diseases. For example, miR-217 was identified as an aging-related miRNA and has been demonstrated to promote atherosclerosis and cardiovascular dysfunction [12]. A previous study identified microRNA-409-3p (miR-409-3p) as an aging-related ncRNA that regulated the senescence of endothelial progenitor [13]. miR-409-3p was also suggested to regulate human cancer progression, such as breast cancer, cervical cancer, and osteosarcoma, and mediate chemotherapy and radiotherapy resistance [14, 15]. Additionally, miR-409-3p was also reported to regulate cardiac fibrosis and angiogenesis, and predict the development of heart failure [16–18]. Therefore, miR-409-3p was speculated to predict postoperative MACE in elderly patients with hip fracture, which was confirmed in the present study.

This study focused on the expression of miR-409-3p in elderly patients received hip fracture surgery. Based on the occurrence of postoperative MACE, the significance of miR-409-3p in predicting patients’ disease development and MACE was evaluated, aiming to provide a potential biomarker for the outcomes of elderly patients with hip fracture.

## Materials and methods

### Study subjects

This is a retrospective study approved by the Ethics Committee of Linfen People’s Hospital. A total of 267 hip fracture patients caused by low-energy trauma and receiving total hip replacement surgery were included, among which there were 104 elderly patients (age ≥ 60 years). The baseline information of study subjects has been summarized in Table S1. All patients have known about the study design and signed informed consent. The inclusion criteria were: 1) patients with neck of femur fracture, femoral intertrochanteric fracture, or subtrochanteric fracture of femur; 2) patients could walk independently or with the aids of tools before fracture; 3) patients received total hip replacement surgery; 4) patients with unilateral closed hip fracture induced by external force. Patients with one of the following items were excluded: 1) patients with a history of acute coronary syndrome, heart failure, or other cardiovascular diseases; 2) patients with pathological fractures; 3) patients with uncompleted clinical records; 4) patients who refuse follow-up surveys or close follow-up. The baseline information was collected from their clinical records.

### Sample collection

Five milliliter fasting venous blood samples were collected from each participant before surgery and centrifugated at 3500 rpm for 10 min to isolate serum. Serum samples were stored at -80°C. The following analysis should be conducted within 48 h of sample collection.

### Real-time quantitative PCR

Total RNA was extracted from serum samples using Trizol reagent (Invitrogen, USA) and evaluated by the ratio of OD260/280 (1.8-2.0). Reversed transcription was performed to generate cDNA with the TaqMan miRNA reverse transcription kit (Thermofisher Scientific, USA). PCR amplification was performed on the LightCycler 480 real-time PCR system (Applied Biosystem, USA) with the help of the SYBR Green kit, according to the manufacturer’s protocol. The relative expression of miR-409-3p was calculated with the 2^-ΔΔCT^ method with U6 as an internal reference.

### Follow-up survey

A 1-year follow-up survey was conducted to summarize the occurrence of MACE in enrolled patients. The occurrence of one of the following events was defined as MACE: all-cause death, postoperative heart failure, postoperative atrial fibrillation, postoperative myocardial infarction, and cardiovascular re-hospitalization. The follow-up data were analyzed by Kaplan-Meier analysis and Cox regression analysis to identify risk factors for the occurrence of MACE in hip fracture patients, especially in elderly patients. There were no missing patients during the follow-up in this study.

### Statistical analyses

A difference comparison between the two groups was performed with a student’s t-test using SPSS 26.0 software. The occurrence of MACE was summarized by the Kaplan-Meier curve. The risk factors for MACE were evaluated by logistic regression analysis and Cox regression analysis. The association of miR-409-3p with patients’ clinicopathological features was estimated by the Chi-square test. *P* < 0.05 indicates statistical significance.

## Results

### Aging is the risk factor for MACE in patients receiving hip fracture surgery

**Fig. 1:**
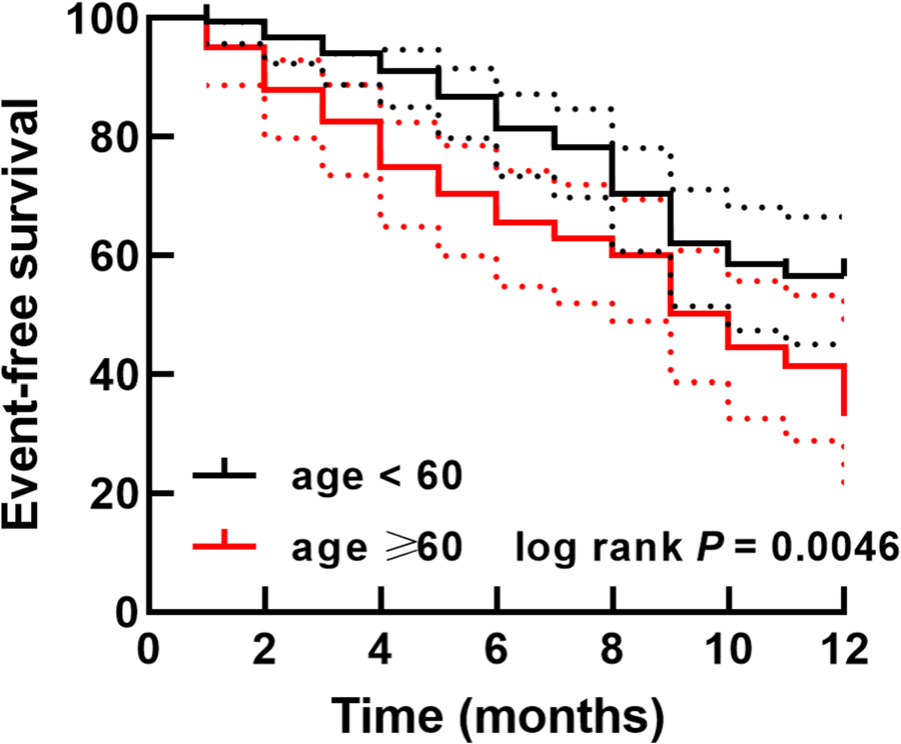
Kaplan-Meier curve of the event-free survival of hip fracture patients based on age. The endpoints were defined as the occurrence of MACE, including all-cause death, postoperative heart failure, postoperative atrial fibrillation, postoperative myocardial infarction, and cardiovascular re-hospitalization with 90 d after surgery

The enrolled patients were aged from 30 to 80 years with an average age of 55.69 ± 14.47 years. There were 104 elderly patients (age ≥ 60 years) included accounting for 38.95% of the study subjects. According to the 1-year follow-up survey, there were 91 occurred MACE after hip fracture surgery in the enrolled patients, including 13 dead cases (14.29%), 17 postoperative heart failure cases (18.68%), 14 postoperative atrial fibrillation cases (15.38%), 22 postoperative myocardial infarction cases (24.18%), and 25 cardiovascular re-hospitalization cases (27.47%). The incidence of MACE between different hip fracture types was not significantly different (data not shown). The results of logistic regression analysis showed that age was a risk factor for the occurrence of MACE (OR = 2.573, 95% CI = 1.277-5.182) together with other renal and cardiac function indexes, RCRI, LVEF, NT-proBNP, CK-MB, and hsTnI (Table 1). Consistently, the elderly patients showed a higher occurrence of MACE than that of patients aged < 60 (Fig. 1, Log-rank *P* = 0.0046).

**Table 1:** Logistic regression analysis evaluating the risk factors for MACE in patients receiving hip fracture surgery

Features	95% confidence index	Odd ratios	*P*-value
Age	1.277–5.182	2.573	0.008
Sex	0.888–3.587	1.785	0.104
Smoking	0.801–3.625	1.704	0.166
Drinking	0.583–2.564	1.222	0.595
RCRI	1.495–6.378	3.088	0.002
LVEF	2.258–13.116	5.442	< 0.001
NT-proBNP	1.732–7.171	3.524	0.001
CK-MB	2.259–9.688	4.678	< 0.001
hsTnI	3.601–15.271	7.415	< 0.001

*RCRI* Revised cardiac risk index, *LVEF* Left ventricular ejection fraction, %, *NT-proBNP* N-terminal pro-Brainin natriuretic peptide, pg/mL, *CK-MB* Creatine kinase-MB, ng/mL; *hsTnI* High sensitivity troponin I, pg/mL

### Upregulated miR-409-3p in elderly patients was associated with the cardiac and renal function of elderly patients

**Fig. 2:**
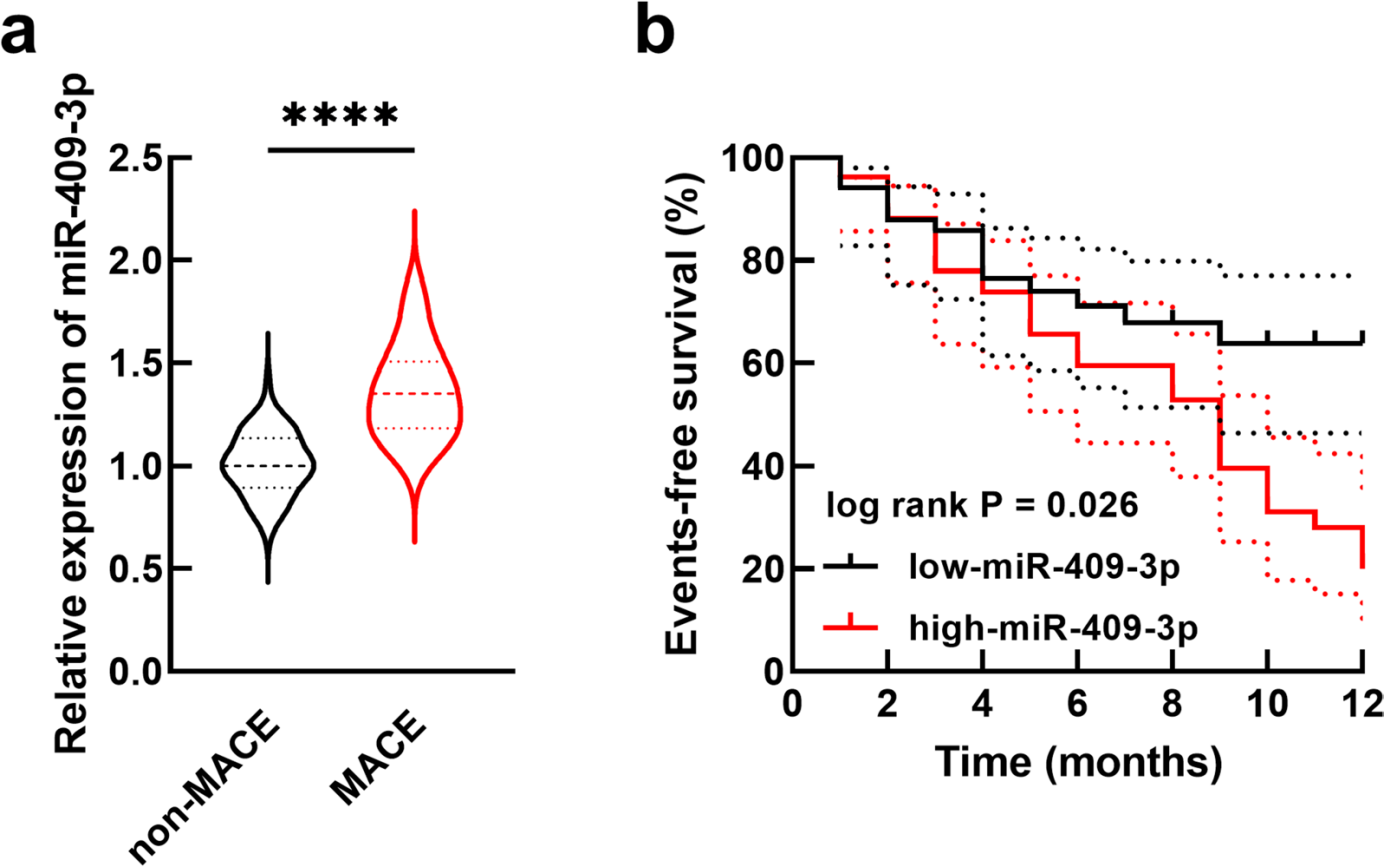
Expression of miR-409-3p in elderly hip fracture patients occurred MACE or not (a) and its correlation with the occurrence of MACE (event-free survival). ****P < 0.0001

In elderly patients, the serum miR-409-3p was significantly higher in patients occurring MACE than in patients without MACE (Fig 2a, *P *< 0.0001). Based on the mean value of serum miR-409-3p (1.40), patients were grouped into the low-miR-409-3p and the high-miR-409-3p group. The higher serum miR-409-3p was associated with the elderly age (*P* = 0.019), the higher level of RCRI (*P* < 0.001), lower LVEF (*P* = 0.008), and the higher levels of cardiac and renal function indexes, NT-proBNP (*P* = 0.011), CK-MB (*P* < 0.001), and hsTnI (*P* < 0.001, Table 2).

**Table 2:** Correlation of miR-409-3p with elderly patients’ clinicopathological features

	Total (*n* = 104)	Low-miR-409-3p	High-miR-409-3p	*P*-value
Age				0.019
< 70	51	31	20	
≥ 70	53	20	33	
Sex				0.198
Male	67	36	31	
Female	37	15	22	
Smoking				0.531
Yes	44	20	24	
No	60	31	29	
Drinking				0.649
Yes	39	18	21	
No	65	33	32	
RCRI				< 0.001
< 3	49	33	16	
≥ 3	55	18	37	
LVEF				0.008
< 50%	31	9	22	
≥ 50%	73	42	31	
NT-proBNP				0.011
< 900	50	31	19	
≥ 900	54	20	34	
CK-MB				< 0.001
< 2.0	48	35	13	
≥ 2.0	56	16	40	
hsTnI				< 0.001
< 7.6	47	34	13	
≥ 7.6	57	17	40	

*RCRI* Revised cardiac risk index, *LVEF* Left ventricular ejection fraction, %, *NT-proBNP* N-terminal pro-Brainin natriuretic peptide, pg/mL, *CK-MB* Creatine kinase-MB, ng/mL, *hsTnI* High sensitivity troponin I, pg/mL

### Upregulated miR-409-3p predicted the occurrence of MACE in elderly patients

Patients in the high-miR-409-3p group were more likely to occur MACE than the patients in the low-miR-409-3p group (log-rank *P* = 0.026, Fig. 2b). Moreover, miR-409-3p was also identified as a predictor for MACE in elderly patients who received hip fracture surgery (HR = 2.454, *P* = 0.008) as well as RCRI (*P* = 0.044), LVEF (*P* = 0.045), NT-proBNP (*P* = 0.031), and CK-MB (*P* = 0.042, Table 3).

**Table 3:** Cox regression analysis evaluating the risk factors for MACE in elderly patients receiving hip fracture surgery

	95% CI	HR	*P*-value
miR-409-3p	1.269–4.744	2.454	0.008
Age	0.714–3.516	1.584	0.258
Sex	0.729–2.679	1.397	0.314
Smoking	0.822–2.902	1.544	0.177
Drinking	0.564–2.055	1.077	0.822
RCRI	1.021–5.054	2.271	0.044
LVEF	1.014–3.897	1.988	0.045
NT-proBNP	1.084–5.293	2.395	0.031
CK-MB	1.030–5.003	2.270	0.042
hsTnI	0.738–4.143	1.748	0.205

*RCRI* Revised cardiac risk index, *LVEF* Left ventricular ejection fraction, *NT-proBNP* N-terminal pro-Brainin natriuretic peptide, *CK-MB* Creatine kinase-MB, *hsTnI* High sensitivity troponin I

## Discussion

MACE is one of the most common postoperative complications of hip fracture repair surgery, which significantly affects the recovery of patients [10, 19]. A previous study reported that the occurrence of heart failure in elderly patients who received hip fracture repair surgery reached up to 16% [20, 21]. However, another study found that the occurrence of MACE was 12% in elderly hip fracture patients [21, 22]. Therefore, the occurrence of MACE might be influenced by research location, time, and other factors. In this study, the occurrence and risk factors of MACE in patients receiving hip fracture repair surgery were investigated, where age was identified as a critical risk factor. The close association between age and MACE has been widely confirmed in previous studies. For example, a recent China research demonstrated that elderly ST-elevation myocardial infarction patients showed a 3.3-time increasing incidence of MACE than younger patients [23]. However, several studies found that the postoperative complications of hip replacement were not related to age, excluding MACE [24]. Additionally, except for age, the cardiac and renal function indexes were also identified as risk factors for MACE. Cardiac insufficiency is the main cause of MACE. On the one hand, perioperative pain, blood loss, and massive fluid rehydration enhance cardiac load and increase cardiac susceptibility. On the other hand, based on the original cardiac dysfunction of elderly patients, cardiac decompensation is more likely to occur [25, 26]. In addition, kidney dysfunction also increases cardiac load resulting in changes in the left atrium and ventricles and therefore induces the cardiac system [27, 28]. Therefore, the prediction of MACE should consider more basic conditions of patients, such as cardiac and renal function, and the significance of age should not be overestimated. It is also necessary to find a biomarker to provide a more accurate prediction of MACE.

Previously, the significance of miRNAs in predicting MACE in various patients has been widely reported. For instance, miR-126 was identified as a marker for indicating the occurrence of MACE in patients receiving percutaneous coronary intervention [29]. miR-146a-5p, miR-223-3p, and miR-142-3p were demonstrated to predict MACE in young patients with acute ST-elevation myocardial infarction [30]. These studies focused on the occurrence of MACE in cardiovascular diseases, there was a lack of biomarkers for predicting postoperative MACE in elderly patients receiving orthopedic surgery [19]. Herein, the significant upregulation of miR-409-3p was observed in elderly patients who received hip fracture repair surgery and occurred MACE. Upregulated miR-409-3p was associated with the elderly age and decreasing cardiac and renal function of patients. Patients with higher serum miR-409-3p levels were susceptible to MACE. miR-409-3p was identified to serve as an age-related miRNA, which is consistent with the results of this study [13]. Additionally, miR-409-3p was revealed to regulate cell progression and mediate cardiovascular and cerebrovascular diseases. The clinical significance of miR-409-3p in screening and monitoring disease development has also been widely noticed. For example, in atrial fibrillation patients, miR-409-3p showed a significant downregulation and was independently associated with disease onset and prognosis [31]. Reduced miR-409-3p was also identified as an indicator of the adverse outcomes of breast cancer patients [32]. The predictive value of miR-409-3p in elderly patients receiving hip fracture repair surgery suggested its potential to serve as a biomarker for MACE.

However, there are still many works need to be done in the future research. Previous extensive studies have demonstrated that early surgery could effectively reduce the occurrence of postoperative complications in patients with hip fracture, and most cases have also been considered emergency surgery [33]. All patients in the present study underwent surgery within 48 h of admission, and the significance of operation timing in predicting MACE was not evaluated. Future studies should include more patients with different surgery timing to clarify its importance in decreasing postoperative complications, especially reducing MACE.

In conclusion, age is a risk factor for the occurrence of MACE in patients with hip fracture. In elderly patients, upregulated miR-409-3p could predict the occurrence of MACE and was associated with patients’ insufficient cardiac and renal function.

### Supplementary Information

**Additional file 1: Table S1.** Baseline information of study subjects.

## Data Availability

The datasets used and/or analysed during the current study are available from the corresponding author on reasonable request.
