# Supplementary material for: Increasing serum miR-409-3p predicts the major adverse cardiac adverse events in elderly patients after hip fracture surgery
Source: BMC Musculoskelet Disord. 2023 Nov 28;24:920. doi: 10.1186/s12891-023-07049-7 (PMC10683352; doi:10.1186/s12891-023-07049-7)
Supplement: Supplementary file 1 — Additional file 1: Table S1. Baseline information of study subjects. [file 12891_2023_7049_MOESM1_ESM.docx]

Table S1. Baseline information of study subjects

|  | Study subjects |
| --- | --- |
| Age (years) | 55.69 ± 14.47 |
| Gender (male/female) | 98/169 |
| RCRI | 1.41 ± 1.70 |
| LVEF (%) | 38.26 ± 8.35 |
| NT-proBNP (pg/mL) | 897.66 ± 118.76 |
| CK-MB (ng/mL) | 2.01 ± 0.33 |
| hs-TnI (pg/mL) | 7.62 ± 1.34 |
| Type of hip fracture (n, %) |  |
| Neck of femur fracture | 109, 40.82 |
| Femoral intertrochanteric fracture | 89, 33.33 |
| Subtrochanteric fracture of femur | 69, 25.85 |

RCRI: revised cardiac risk index; LVEF: left ventricular ejection fraction; NT-proBNP: N-terminal pro-Brainin natriuretic peptide; CK-MB: creatine kinase-MB; hsTnI: high sensitivity troponin I.
